# Nonvolatile Reconfigurable Transistor via Ferroelectrically Induced Current Modulation

**DOI:** 10.1021/acsami.4c16400

**Published:** 2025-02-06

**Authors:** Daniele Nazzari, Lukas Wind, Masiar Sistani, Dominik Mayr, Kihye Kim, Viktor Wahler, Walter M. Weber

**Affiliations:** Institute of Solid State Electronics, Technische Universität Wien, Gußhausstraße 25-25a, 1040 Vienna, Austria

**Keywords:** Ferroelectric Hf_0.5_Zr_0.5_O_2_ (HZO), Reconfigurable Field Effect Transistor (RFET), Nonvolatile reconfigurability, Charge injection modulation, Logic in Memory (LiM), Multilevel operation

## Abstract

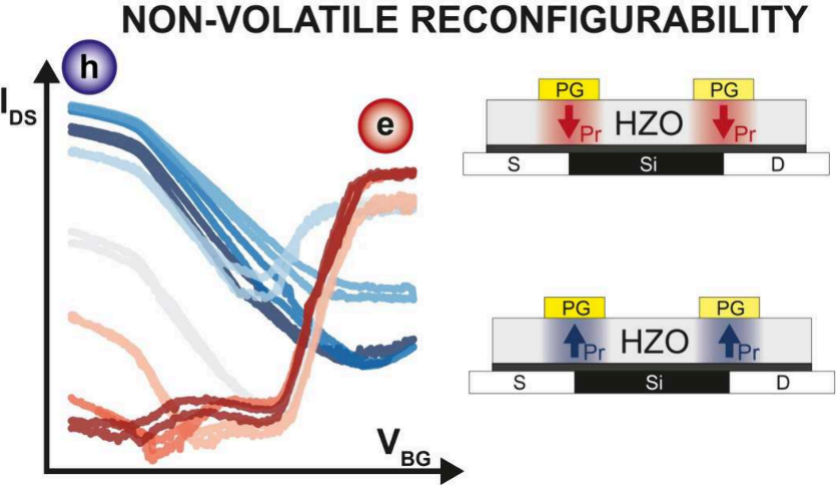

New logic in memory (LiM) architectures, able to unify memory and logic functionalities into a single component, are highly promising for executing self-learning algorithms such as artificial neural networks (ANNs), with lower energy requirements. The multigated reconfigurable field effect transistor (RFET) is a novel type of logic device that can be fully reconfigured at run-time, promising to be a very versatile platform for logic applications. If equipped with a memory element, then it would represent the ideal building block for LiM-enabling hardware with embedded self-learning capabilities. To reach this goal, here we investigate the integration of a ferroelectric Hf_0.5_Zr_0.5_O_2_ (HZO) layer onto dual top gated RFETs. We demonstrate that HZO polarization charges can be successfully employed to tune the height of the two Schottky barriers, influencing the injection behavior and allowing the selection of the majority carriers, thus defining the transistor mode and switching it between a fully p-type transport to a prevalently n-type one. Moreover, we show that the modulation strength is strongly dependent on the height of the pulse used to polarize the ferroelectric domains, allowing for the selection of different current levels. All the different achievable states show a good retention over time, owing to the stability of the HZO polarization. The limitations of the produced devices are discussed, alongside possible mitigation strategies. The presented results demonstrate that ferroelectric HZO can modulate the carrier injection across Schottky barriers in RFET devices. This approach paves the way for the future realization of a fully optimized nonvolatile RFET, an ideal building block for novel LiM hardware, enabling low-power circuits for ANN execution.

## Introduction

Deep learning algorithms have recently demonstrated extraordinary success, rapidly becoming ubiquitous.^1^ However, energy requirements and carbon emissions for training and operating artificial neural networks (ANNs) are on a steep increase and will soon reach unsustainable levels, given the current hardware.^2,3^

In modern computing, a great deal of resources are spent moving data from processing units to memory and back, strongly impacting data-intensive applications such as ANNs and ultimately limiting the computational performances, a problem better known as von Neumann bottleneck.^4^ Energy efficiency, however, can only be marginally improved through further scaling of the electronic components, as this process is becoming ever increasingly complex and costly, leading to a clear departure from the exponential trend that was well described by Moore’s law.

New logic-in-memory (LiM) architectures have been proposed as a possible solution to this problem.^5^ These models aim at drastically reducing data transfer by relying on computing units that are also able to locally store information.^6−8^ Over the recent years, LiM has been experimentally demonstrated recurring to classic charge-based^9,10^ or resistance-based memories,^6,11,12^ as well as innovative memory concepts based on 2D material heterostructures,^13^ ferroelectric field effect transistors,^14^ and photonic platforms.^15^

In general, a successful LiM-enabling technology must fulfill the fundamental requirement of being able to reach a high density of components per unit area. This can be achieved by combining scaling with a smart choice of device architecture: while the former option is limited by rising costs and complexity, the latter approach has been shown to hold great potential for the realization of area-optimized logic circuits.

Within this framework, the recent development of a reconfigurable field effect transistor (RFET) has introduced a device in which electrical behavior is not fixed at production but can be freely programmed to be either n- or p-type.^16^ This flexibility is achieved through the addition of two polarity gate metal contacts, to tune the Schottky barriers forming between the source (S) and drain (D) contacts and the semiconducting channel. This allows one to filter the charge carriers, selecting a hole- or electron-based transport. Despite the small penalty introduced by the marginal increase in the single transistor complexity, RFETs offer a large advantage through reconfigurability, allowing for the realization of adaptable circuits based on polymorphic logic gates,^17^ with an important area reduction compared to the standard CMOS architecture.^18^

Thus, it is clear that this innovative architecture represents a promising building block for the realization of adaptable, highly dense, LiM-enabling hardware, if equipped with a nonvolatile memory element. To introduce this functionality, here we investigate the integration of a Hf_0.5_Zr_0.5_O_2_ (HZO) ferroelectric layer, localized precisely below the polarity gates (PGs) of a silicon-on-insulator (SOI)-based RFET. Similar approaches, albeit not restricting the ferroelectric influence to the Schottky regions only, have been proven viable for the realization of a nonvolatile ferroelectric memory^19^ and artificial synapses.^20,21^

In this work, we show that by applying programming pulses to the PGs, it is possible to control the magnitude and direction of the ferroelectric polarization. In this way, the Schottky barriers that regulate carrier injection can be tuned by the remnant polarization (P_*r*_) charges without the need for applying an external voltage. Thus, when the ferroelectric domains are pinned in a specific polarization state, the resulting transport configuration is maintained over time. Multiple different states can be achieved by tuning the height of the programming pulse, showing a stable separation over the chosen time window. In addition to a precise characterization of the behavior of the engineered devices, a thorough discussion regarding their current limitations is also presented, with a focus on some viable strategies for their improvement.

The data presented in this work demonstrate that ferroelectric HZO can be successfully employed to modulate carrier injection over Schottky barriers. This approach is promising for the future realization of a fully optimized nonvolatile reconfigurable field effect transistor.

## Results and Discussion

**Figure 1:**
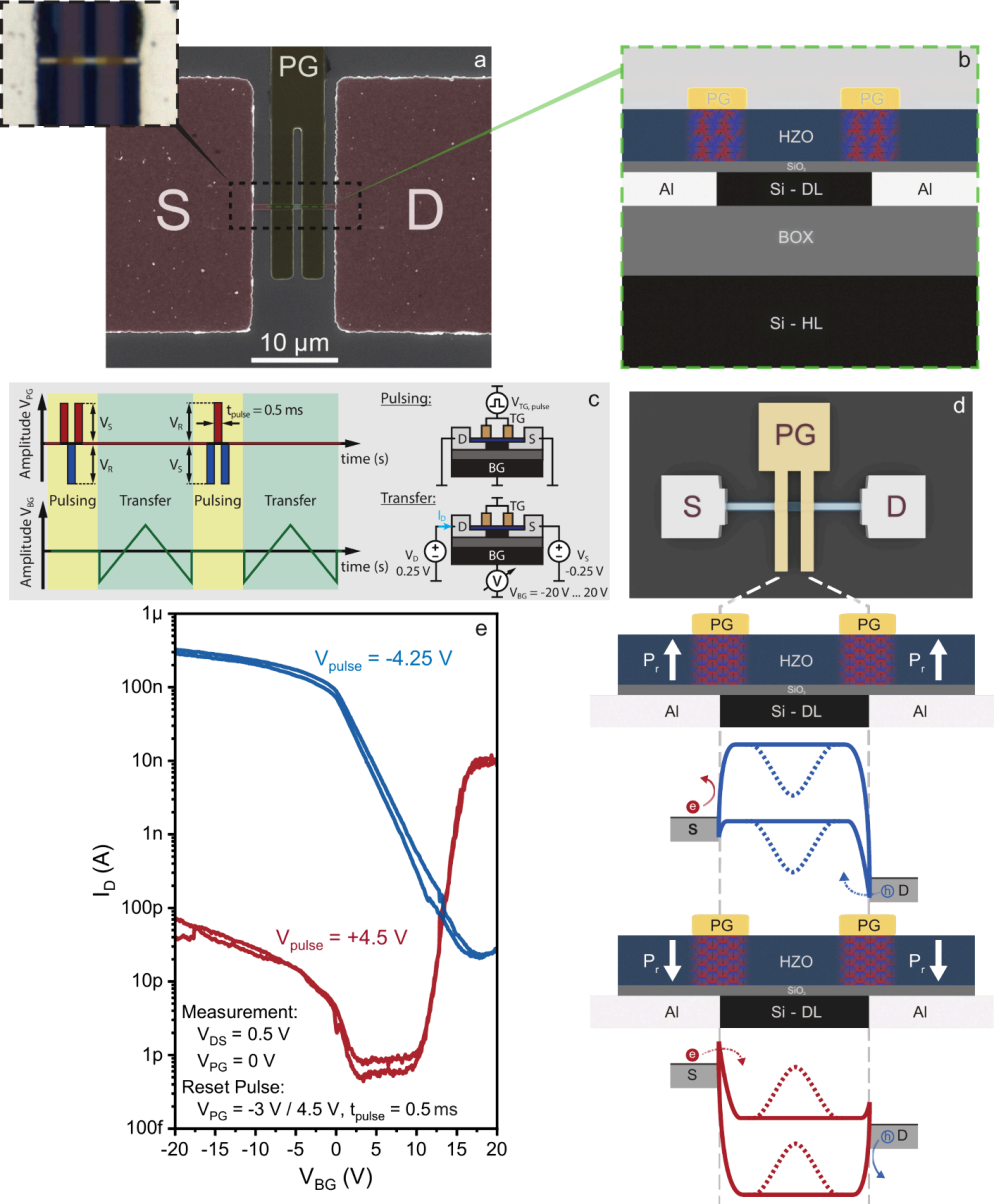
a) Scanning electron microscope image of one of the realized device. Labels identify the source (S), drain (D), and polarity gate (PG) contacts. The inset shows an optical microscope image of the same device. The Al–Si interfaces can be identified below the transparent TiN PG gates. b) Cross-sectional representation of the realized stack. The HZO layer ferroelectric domains are located only under the PG area. c) Adopted pulsing and transfer measurement schemes, alongside the associated device configurations. d) Schematic representation of the band structure in the Si segment, with emphasis on the Schottky barrier configuration. Depending on the direction of the remnant polarization vector (Pr), the barriers are influenced to block the injection of electrons (blue) or holes (red). e) Transfer characteristics obtained by sweeping the back gate of the device after a polarization sequence has been applied. Respectively, the blue curve is obtained after a pulse height of −4.25 V is applied, while the red curve is obtained with a pulse height of +4.5 V. The curves are associated with the band structures of the same color depicted in panel d.

Figure 1a and b show a SEM image of the realized device, with the highlighted metallic terminals (PG, S, D) alongside an inset taken with an optical microscope and a schematic representation of the material stack. As described in the Experimental Section, the devices are based on a commercial SOI substrate, with a device layer thickness of 20 nm on top of a 100 nm thick buried oxide (BOX) and on a Si handling layer, which is used as the back-gate (BG). Once the Si nanosheet structures are defined through laser lithography and reactive ion etching, the dielectric stack is grown following a two-step process. First, a 0.9 nm thick SiO_2_ layer is grown via chemical processing, followed by the deposition of an amorphous 8.5 nm thick HZO layer via ALD. The importance of the thin SiO_2_ layer is clarified later in the discussion. Once the dielectric stack is completed, the metallic contacts are defined using laser lithography. The dielectric layer is removed via Ar^+^ sputtering, followed by Al sputtering to form the two S and D contacts. The dual PGs are defined by sputtering TiN, after an additional lithographic step. More details on the fabrication process can be found in the Experimental Section. Once the stack is completed, a rapid thermal annealing step is performed, with the 2-fold purpose of enabling the Al–Si exchange reaction^22^ and the crystallization of the HZO layer.^23^ This causes Al to diffuse along the Si nanosheet, reaching the area below the two fingers of the PG structure. Thanks to the transparency of the thin TiN layer, the two interfaces formed between the Si segment and the diffused Al are clearly visible below the PG, as shown in the inset of Figure 1a. The precise position and sharpness of both Al–Si junctions below the PG are essential to ensure a good electrostatic control of the Schottky barriers, fundamental for the functionality of the devices. The channel lengths are measured to be 3.9 ± 0.9 μm. The variability is presumably due to local changes in the temperature and etch rates. It must be noted that classic (i.e., volatile) RFETs are expected to operate without degradation for channel lengths as small as 8 times the screening length, as demonstrated via TCAD simulations.^24^

As mentioned earlier, the annealing process triggers the crystallization of the amorphous HZO layer, giving rise to different crystalline phases depending on which material is interfacing the oxide layer. The regions of HZO that are in contact with TiN are subjected to an in-plane stress that leads to the formation of a ferroelectric orthorhombic phase, in a mix with other non-ferroelectric crystalline structures. Differently, the material not in contact with TiN crystallizes only in the non-ferroelectric phases, giving rise to a trivial dielectric layer.^25^ As summarized in Figure 1b, the two ferroelectric segments are therefore located between the two TiN fingers and the Al–Si interfaces, making it possible to control the ferroelectric remnant polarization via the PGs. At the same time, the Schottky barriers at the Al–Si interfaces can be strongly influenced by the HZO remnant polarization, as a consequence of their extreme vicinity, determining the overall electrical behavior of the device.

To obtain the data presented in this work, only two different electrical operations are performed, denoted “SET” and “READ”, schematically represented in Figure 1c. As the name suggests, the “SET” operation is used to change the polarization state of both ferroelectric segments. This is done by pulsing a voltage of sufficient height (*V*_*PULSE*_) to the PG, while all other terminals are kept grounded. The “READ” operation, instead, corresponds to measuring *I*_*DS*_ while sweeping the back-gate potential (*V*_*BG*_) within values low enough to not trigger a change in the HZO polarization state. As clearly indicated in the scheme, during this operation, the PG terminals are fixed at 0 V. This is different from the normal operation of a classic, volatile RFET device, where the polarity is imposed by applying a fixed voltage at the PG. In the current work, however, the net electric dipole of the aligned ferroelectric domains is able to modulate the Schottky barriers at the Al–Si interfaces in a nonvolatile manner, determining the preferential injection of holes or electrons without the need of applying, respectively, a negative or positive external voltage to PG. The energy landscapes for the two described states are shown in Figure 1d. When a sufficiently negative *V*_*PULSE*_ is applied onto the PG, the ferroelectric domains align to form a dipole oriented toward the PG contacts, resulting in a net negative charge in the vicinity of the semiconductor interface, as shown in the upper portion of the scheme. This determines a local rise in energy of the bands, which leads to a modification of the Schottky barriers in a way that enables hole tunneling, while impeding electron injection. During the “READ” operation, *V*_*BG*_ is swept in a way that enables the flow of charges, as depicted in the band scheme with a solid line, or that blocks it, as indicated by the dashed line. It must be noted that the Schottky barriers at the Al–Si interfaces are almost totally influenced by the HZO layer, as these regions are extremely sharp and close to the ferroelectric segments. While it is true that *V*_*BG*_ affects the whole device, interface regions included, its effect on the HZO segment is negligible, thanks to the thickness of the buried insulation layer. This can be deduced from the fact that only minor changes can be observed between the currents measured during the forward and backward sweeps, indicating no change in the remnant ferroelectric polarization. The “READ” operation results in the measurement of a p-type transfer characteristic, as shown in blue in Figure 1e, as a result of the ferroelectric-mediated band-bending. On the contrary, with the positive and sufficiently high *V*_*PULSE*_, the ferroelectric dipole is oriented in the opposite way, resulting in a net positive charge in the vicinity of the semiconductor interface, as shown in the bottom part of the scheme. This leads to the lowering of the bands in correspondence with the Al–Si interface regions, enabling a preferential injection of electrons, resulting in a more n-type-oriented transfer characteristic, as shown in red in Figure 1e. Clearly, the two states produce strongly different outcomes, with defined on and off regions. The measured transfer characteristics always show an asymmetric behavior between the two states with higher *I*_*ON*_ on the p-side. In addition to this, the threshold voltage of both curves is clearly shifted toward high positive *V*_*BG*_ values. The latter observation is normally explained by the presence of a certain amount of negative fixed charges near the semiconducting channel.^26^ At the same time, an abundance of negative charges could determine an upward band-bending at the Al–Si interfaces, screening the effects of the ferroelectric polarization and determining the preferential injection of holes, thus explaining also why the devices are leaning toward a p-type behavior. The presence of fixed charges is a well-known characteristic of the high-k/Si systems,^27−30^ determined by a wide range of causes.^31−34^ In the specific case of HfO_2_ and ZrO_2_, most trap states are related to the presence of defects in the layer, with an important role taken by oxygen vacancies and oxygen interstitials.^34−36^ First-principle calculations demonstrate that trap levels associated with oxygen vacancies lie close to the Si gap, while those caused by oxygen interstitials are located deep below the Si valence band edge. This means that the latter may not act as a carrier trap but could, instead, represent a source of negative fixed charge.^34,36^

Generally speaking, the trappy interface between high-*k* dielectrics and Si can seriously influence the transport behavior in different ways.^37−39^ First of all, a high concentration of interface traps can drastically degrade the carrier mobility and increase the hysteresis of a device, limiting its performances. Second, due to the brief but extreme band bending taking place during a “SET” operation, even the deep trap states become accessible to the carriers, getting charged for a long period of time. This is detrimental to the expected operation of the device, as the electric field induced by charge trapping is always opposed to the ferroelectric-associated one, thus, reducing or even nullifying its control over the Schottky barriers. To minimize this problem, a thin SiO_2_ layer (*t* ∼ 0.9 nm) is formed at the Si surface by chemical treatment, as described in the Experimental Section, prior to the deposition of the HZO layer. This helps to obtain a very low hysteresis, as seen from the dual-sweep transfer characteristics of Figure 1e. The thickness of the interface oxide is kept as low as possible to maximize the influence of the ferroelectric dipole on the Schottky barriers. At the same time, however, this choice has the disadvantage of keeping the high fixed charges close to the semiconducting channel, producing the effects described above. Nevertheless, due to the extreme thinness of the interface layer, it is reasonable to assume that charges can tunnel through it, even at low *V*_*PULSE*_. It must be stressed that this mechanism of charge injection is of great importance for stabilization of the ferroelectric effect, as it was clearly demonstrated through experiments and simulations. In particular, it was shown that the injection and subsequent trapping of charges in the vicinity of the ferroelectric layer is a fundamental prerequisite for enabling polarization switching in ferroelectric tunnel junctions (FTJs)^40^ as well as in ferroelectric field effect transistors (FeFETs).^41^ Moreover, charge trapping at the ferroelectric interface improves the retention of the spontaneous polarization^42^ but can also limit the achievable currents.^43^ Thus, the density of trap states at the semiconductor–dielectric and dielectric–ferroelectric interfaces, together with the amount of charges injected through the SiO_2_ layer, are parameters that can greatly influence the device behavior. It is, however, critically important for the correct behavior of the device to control the amount of trapped charges, as their excess can negatively impact the endurance of a device.^44^ This type of investigation, however, exceeds the scope of this article and will be addressed in the future.

Overall, the produced devices are all able to achieve clearly separable states, which are, as we will later show, very stable over time, a fundamental requirement for the conceived use. It is very important to notice that the two states shown in Figure 1e are characterized by a current ratio larger than 3 orders of magnitude for *V*_*BG*_ = 0 V, a very important parameter for exploiting the device memory capability in the most efficient possible way.

**Figure 2:**
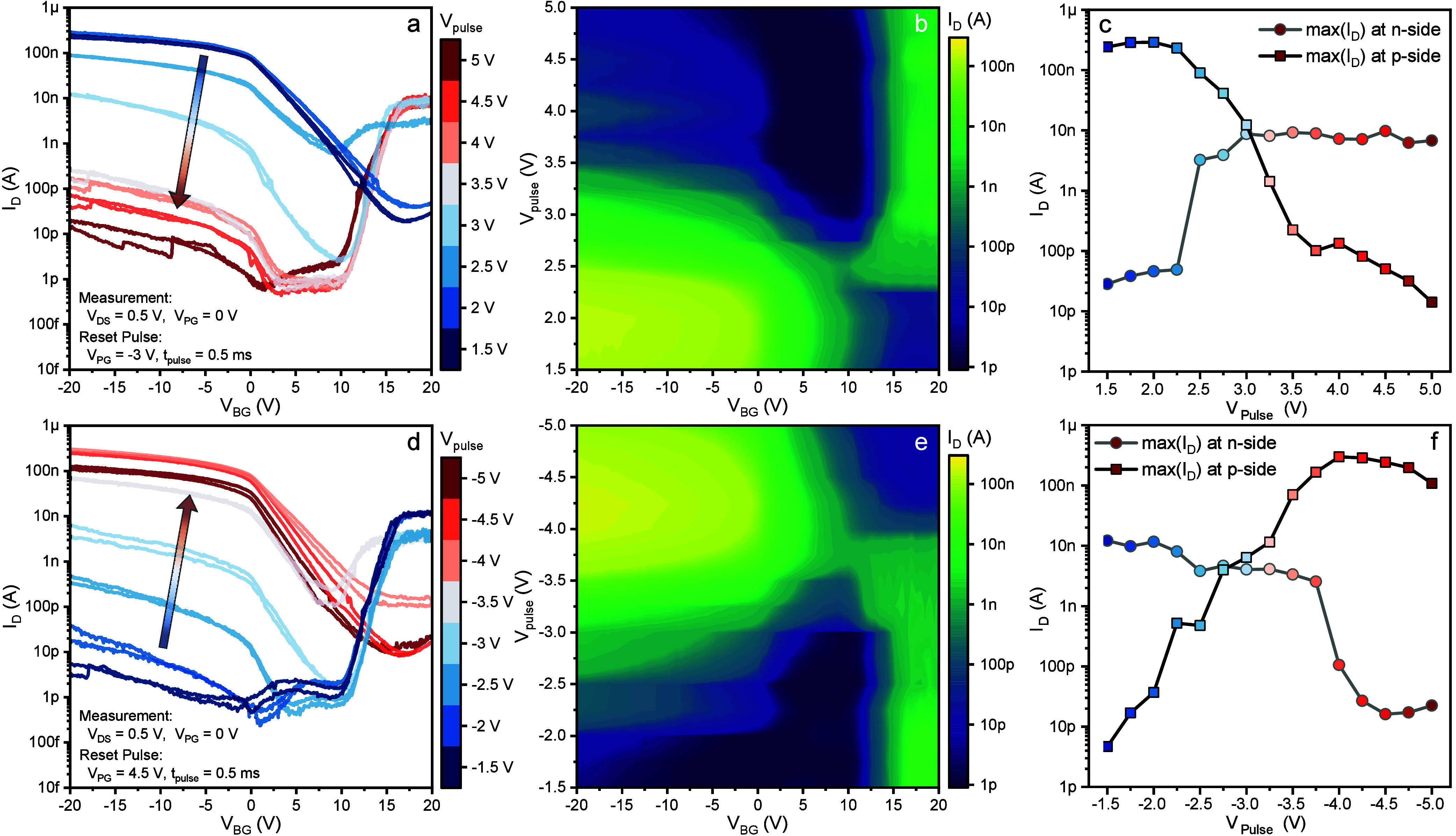
a) Transfer characteristics obtained after applying positive pulses of varying magnitudes to the polarity gate. From blue to red, the magnitude of the applied pulses increases from +1.5 V to +5 V. b) The color map shows the measured drain current as a function of the back gate voltage and of the chosen pulse height, clearly indicating a transition from p- to n-type behavior. c) The maximum drain currents at the n-side (*V*_*BG*_ = +20 V) and at the p-side (*V*_*BG*_ = −20 V) are plotted with respect to the pulse height, showing an exponential modulation. d–f) Same type of plots as in a,b,c but for negative polarization pulses.

Before discussing the stability of the devices, we first focus on investigating the response to different *V*_*PULSE*_ magnitudes. Figure 2a shows different transfer curves obtained after programming the device using an increasingly higher positive *V*_*PULSE*_. After each measurement, the device is reset by applying a sufficiently high reverse *V*_*PULSE*_ of −3 V. Therefore, we always investigate the transition from an almost ambipolar state to an n-type one. The device starts to show a behavioral change when *V*_*PULSE*_ = +2.5 V is used, with a strongly increasing *I*_*n*_ and a slightly decreasing *I*_*p*_. This denotes that the barriers are already changed by the HZO influence, with an increase in probability for electron injection and a slightly higher blockage for holes. Thus, a mixture of carriers can be injected into the semiconducting channel, therefore limiting the capability of *V*_*BG*_ to efficiently block the transport, explaining why *I*_*OFF*_ is still very high. When *V*_*PULSE*_ reaches +3.5 V, a more drastic change is observed, with a strong reduction of *I*_*p*_. By further increase of the amplitude of *V*_*PULSE*_, a stronger reduction of *I*_*p*_ takes place. *I*_*n*_, differently, slightly increases for *V*_*PULSE*_ = +4.5 V, while marginally decreasing when *V*_*PULSE*_ reaches +5 V. It is important to notice that the measured curves do not shift horizontally, showing that the polarization charges are accumulating, as expected, only at the Al–Si interfaces. The color-map on the right (Figure 2b) shows in a more immediate way the change of *I*_*DS*_(*V*_*BG*_) as a function of *V*_*PULSE*_ magnitude. As described earlier, by increasing *V*_*PULSE*_, *I*_*p*_ decreases starting from +2.5 V, while *I*_*n*_ increases, until a *V*_*PULSE*_ of +4.5 V is reached. This observation can be explained by considering different effects: first, it is well-known that the remnant polarization of a ferroelectric layer is limited in magnitude and cannot surpass the value achieved when all the domains are aligned parallel to each other. Therefore, the influence of the ferroelectric layer over the Schottky barriers will not increase indefinitely, but will reach a maximum level at a certain *V*_*PULSE*_. In addition to this, it must also be taken into consideration that, when a “SET” operation with a very high *V*_*PULSE*_ is performed, the band bending at the interfaces is quite extreme, possibly letting some charges tunnel through the SiO_2_ interface layer and occupy certain deep traps. This implies that for an increasing *V*_*PULSE*_ magnitude, the ferroelectric effect will be reduced by the competing trapping field. Moreover, extreme *V*_*PULSE*_ magnitudes can damage the oxide layer, increasing leakage, and thus hindering the ferroelectric response. Figure 2c shows the maximum *I*_*D*_ currents extracted from the p-side (*V*_*BG*_ = −20 V) and n-side (*V*_*BG*_ = +20 V) of the plots shown in Figure 2a. As described previously, the p-side current is suppressed with increasing *V*_*PULSE*_, while the n-side follows the opposite behavior. Clearly, the increase and suppression of the currents follow an exponential trend as a function of applied *V*_*PULSE*_. Nevertheless, the maximum change observed on the n-side is smaller, with an increase of 2 orders of magnitude, compared to the 4 orders of magnitude observable on the p-side.

Figure 2d shows the transfer characteristics obtained after programming the device using an increasingly large negative *V*_*PULSE*_, from −1.5 V to −5 V. In a similar fashion, the device shows drastic changes when the pulsed voltage is larger than −2.5 V, with both a strong decrease of *I*_*n*_ and an increase of *I*_*p*_. Once again, if *V*_*PULSE*_ is too large, carriers can tunnel through the SiO_2_ layer and get trapped in deep levels at the high-*k* interface. In this case, as also noticeable from the color-map on the right side, the reduction of *I*_*p*_ is very evident for *V*_*PULSE*_ < −4.5 V. This stronger trapping effect for holes could be determined by the fact that, as mentioned before, the HZO defects are acting like donors, thus repelling electrons but favoring holes. Overall, the analysis presented in Figure 2 clearly shows that different intermediate states can be obtained by carefully choosing *V*_*PULSE*_, resulting in defined and well-separated values of *I*_*DS*_ from which to choose. Once again, a color-map is shown in Figure 2e, to clearly represent the change of *I*_*DS*_(*V*_*BG*_) as a function of *V*_*PULSE*_ magnitude. The maximum *I*_*D*_ currents from the p-side and n-side are reported in Figure 2f for increasingly negative *V*_*PULSE*_ levels. From this figure, an exponential modulation of the current is observable. Clearly, the peak reached on the p-side is slightly reduced for the largest values of *V*_*PULSE*_ due to the trapping mechanism that was addressed just above.

**Figure 3:**
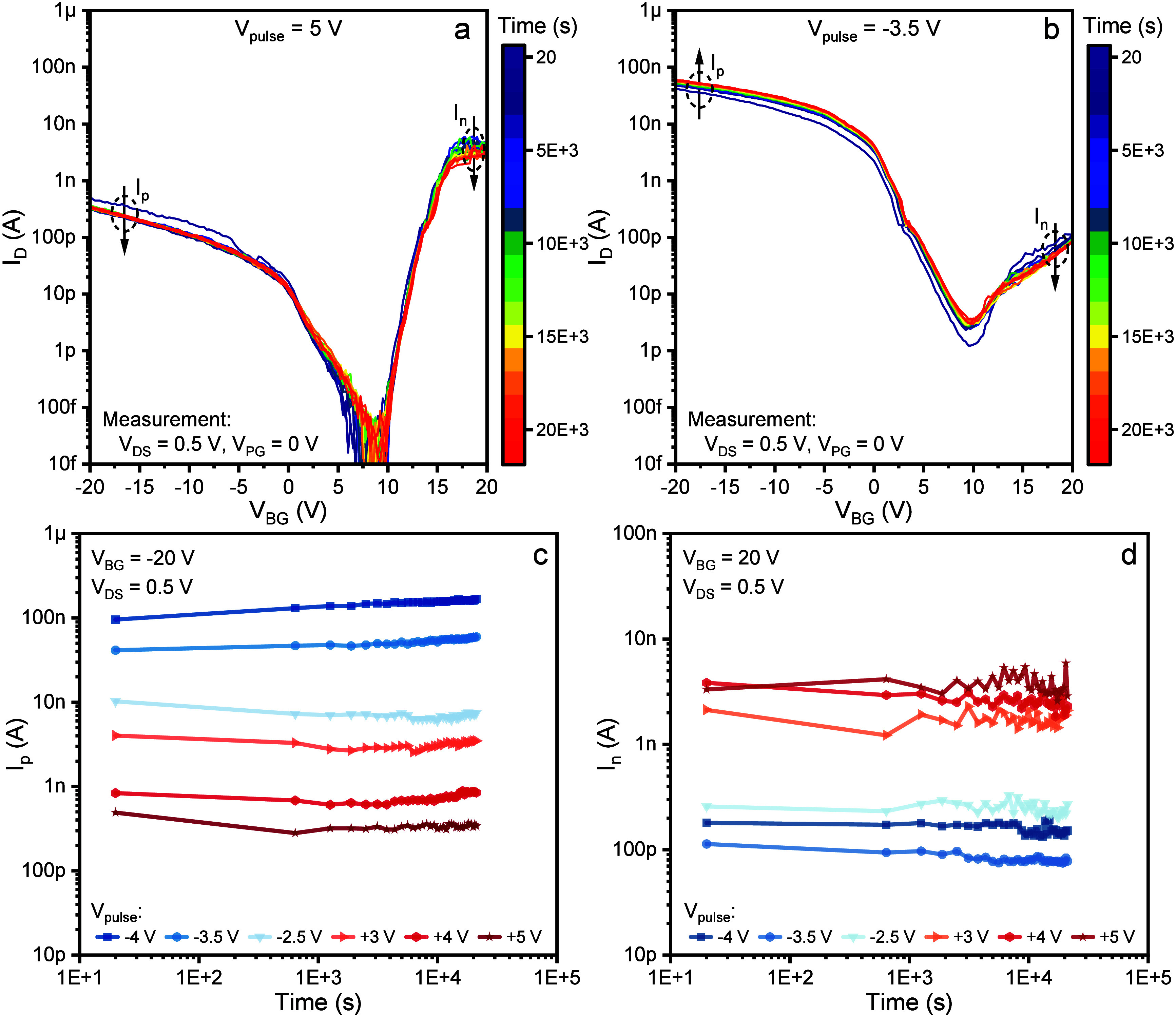
a) The device is polarized using a pulse height of +5 V. The panel reports several transfer curves measured every 15 min, up to 6 h, showing little change. b) Same as a, but after polarizing the device with a pulse height of −3.5 V. c) Time-dependency of the drain currents measured at the p-side (*V*_*BG*_ = −20 V) for different polarization pulse heights. d) Time-dependency of the drain currents measured at the n-side (*V*_*BG*_ = +20 V) for different polarization pulse heights.

A very important characteristic of the developed devices is their observed stability over time. Figure 3 shows the data acquired over a period of ∼6 h. It must be noted that the data shown in Figure 3 are obtained using a different device compared with the previous plots. This was necessary in order to observe the behavior of a pristine device, as the previously used one was affected by a certain amount of charge trapping due to the application of a high *V*_*PULSE*_, as shown in Figure 2. In order to perform the measurements shown in Figure 3, the device is set into one of the states described earlier, by performing a “SET” operation with either +5 V or −3 V *V*_*PULSE*_. After the device is set into a specific state, its transfer characteristic is measured every 10 min. In between measurements, the device is kept connected to the probe station with grounded voltages. The measured data are shown in Figure 3a and b. Clearly, the device shows remarkable stability within the probed time window, with only minor changes in the *I*_*ON*_ and *I*_*OFF*_ or the *I*_*n*_/*I*_*p*_ ratio. This shows that almost no depolarization happens during this time, keeping the influence of the ferroelectric layer on the barriers unchanged. An interesting parameter to track over time is represented by the *I*_*ON*_ for different *V*_*PULSE*_ levels. This is reported in Figure 3c and d for *I*_*ON*_ at *V*_*BG*_ = −20 V (*I*_*p*_) and at *V*_*BG*_ = 20 V (*I*_*n*_), respectively. Similarly to what was observed in Figure 2, different levels of *I*_*n*_ and *I*_*p*_ can be observed as a result of certain choices for *V*_*PULSE*_. Importantly, all of the observed states are similarly stable over time, keeping a clear separation through the whole measurement window. However, as time passes by, *I*_*p*_ shows a slightly upward trend, especially marked for negative values of *V*_*PULSE*_. This behavior is in contrast with the expected decrease of the ferroelectric effect over time due to the onset of depolarization. Despite not having grasped yet a full explanation for the observed behavior, we speculate that this is the result of a competition between the electrostatic effect of trapped charges and ferroelectric polarization. As observed in Figure 2, charge trapping can occur during the “SET” operation due to the extreme band bending, allowing charges to tunnel through the SiO_2_ interface and to occupy empty levels. Thus, at the beginning of a retention test, the Schottky barriers feel the combined influence of these two effects, which are opposing each other, as explained earlier. As time passes, the trapped charges are partially released at a rate faster than that of the depolarization of the ferroelectric layer, thus resulting in a perceived increase of the ferroelectric effect. This, however, is only visible for *I*_*p*_, implying that the trapping of holes is more favorable, in accordance with the earlier argumentation.

## Conclusions

In this work we have presented the fabrication and characterization of a nonvolatile, SOI-based, reconfigurable field effect transistor where an HZO ferroelectric segment is precisely placed above the metal–semiconductor Schottky barriers. This design choice allows us to investigate the impact of the ferroelectric polarization onto the carrier injection across the metal–semiconductor barriers without altering the transport properties of the semiconducting channel. Importantly, the HZO layer is integrated above an ultrathin SiO_2_ layer, enabling transfer characteristics with very low hysteresis. We have shown that, by applying a sufficiently high negative or positive voltage to the dedicated program gates through a pulsing sequence, it is possible to successfully modulate the carrier injection, changing the observed behavior from unipolar p-type to predominantly n-type and back, without requiring the application of an external voltage. The observed changes are proof of a ferroelectric modulation of the Schottky barriers, clearly ruling out charge-trapping as the responsible mechanism. We have systematically investigated the device behavior as a function of the program pulse height, showing that minimum voltages of −4 V and +3.5 V are needed in order to fully change the transfer characteristic to, respectively, p-type and n-type. The choice of intermediate pulse voltages results in different current levels, i.e., a multitude of well-separated states. Too high of pulse voltages, however, can induce charge trapping, which determines a weakening of the ferroelectric influence. The examined devices show high stability over time, retaining the stored configuration for at least 5 h. This work paves the way for fully optimized nonvolatile reconfigurable field effect transistors, a promising building block for scaled, low-power hardware combining logic and memory capabilities.

## Experimental Section

### Device Fabrication

The devices have been realized following a slightly modified version of the well-established Al–Si RFETs fabrication strategy developed by our group for the realization of highly reliable reconfigurable transistors.^17,45^ The devices are obtained starting from an industrial SOI substrate with a 20 nm thick, lightly p-doped (B, ∼10^15^cm^–3^) Si device layer in (100) orientation on top of 100 nm thick buried SiO_2_ (BOX) and a 500 μm thick, low doped Si substrate. The ∼500 nm wide Si nanosheets were patterned using laser lithography and an SF_6_-O_2_-based dry etching process. After dipping the substrate in buffered hydrofluoric acid (HF:NH4F = 1:7) to remove the native oxide, the 0.9 nm thick interface SiO_2_ layer was chemically grown following the RCA-2 procedure (H_2_O:HCl:H_2_O_2_ (6:1:1) at *T* = 343 K for 10 min). The 8.5 nm thick HZO layer (Hf_0.5_Zr_0.5_O_2_) was grown by ALD at *T* = 523 K using TEMAHf, TEMAZr as precursors, and H_2_O as oxidant source, with N_2_ as the carrier gas. Initially, two cycles of ZrO_2_ were deposited on the SiO_2_ interface, succeeded by 68 supercycles of alternating TEMAHf and TEMAZr process cycles. The S/D pads were patterned by laser lithography, followed by Ar^+^ (at a power of 100 W) sputtering to remove the dielectric layer in the contact area and subsequent Al sputter deposition (100 nm) and lift-off techniques. The 50 nm thick TiN top gates (PGs) were then defined by laser lithography and reactive sputtering of Ti in N_2_ plasma at 100 W, with an N_2_ flow of 6 sccm and at a working pressure of 6 × 10^–3^ mbar, followed by lift-off. Several steps of rapid thermal annealing (RTA) at *T* = 773 K in a N_2_ atmosphere were performed to crystallize the HZO into the ferroelectric orthorhombic phase and simultaneously induce the Al–Si exchange reaction to achieve the desired Si channel lengths, with both Al–Si interfaces aligned below the PG.

### Electrical Measurements

The electrical measurements were performed using a Keysight B1500A semiconductor analyzer in combination with a Lakeshore PS-100 probe station in ambient air. The SET operation is performed by pulsing the PG terminals while keeping all of the other connections grounded. The device is always reset before the PG terminal is pulsed with the chosen voltage. The READ operation is performed by sweeping the BG terminal while reading the current flowing between the S and D terminals, while keeping PG grounded. All pulses had a length of 0.5 ms.
